# Comparison Between Single Anastomosis Duodeno-Ileal Bypass with Sleeve Gastrectomy (SADI-S) and Roux-En-Y Gastric Bypass (RYGB) in Terms of Weight Loss, Associated Medical Problems Remission, and Complications: A Systematic Review with Meta-Analysis

**DOI:** 10.1007/s11695-025-08092-0

**Published:** 2025-07-22

**Authors:** Lucas Sabatella, Daniel Aliseda Jover, Patricia M Ortega, Manuel Fortún Landecho Acha, Fernando Rotellar Sastre, Carlota Tuero Ojanguren, Nuria Blanco Asensio, Adriana Uriz Pagola, Victor Valentí Azcárate

**Affiliations:** 1 Clínica Universidad de Navarra, Pamplona, Spain; 2 Imperial College Healthcare NHS Trust, London, United Kingdom

**Keywords:** SADI-S, RYGB, Weight loss, Associated medical problems remission, Postoperative outcomes

## Abstract

**Background:**

Evaluate and compare the results of weight loss outcomes, associated medical problems resolution, and complications in the short and long term between SADI-S and RYGB.

**Methods:**

A systematic review was conducted following PRISMA guidelines. Studies comparing SADI-S and RYGB as primary surgery were included if they reported weight loss (total weight loss (TWL), excess weight loss (EWL), or body mass index (BMI) changes), associated medical problems remission (diabetes and hypertension), and postoperative outcomes (complications, hospital stay, and operative time). A meta-analysis of mean differences (MD) was conducted to assess continuous outcomes, and a meta-analysis of odds ratios (OR) was performed to evaluate the categorical variables; a random effects model was used.

**Results:**

Eight studies, including 4259 patients (1625 SADI-S; 2634 RYGB), were analysed. Six studies with over 2 years of follow-up (mean 3.93 years (1.79)) were included for long-term outcomes, while all eight were considered for short-term outcomes. SADI-S resulted in a statistically significant higher total weight loss (MD 10.03; 95% CI 4.7–15.35; *p* < 0.001), excess weight loss (MD 10.15; 95% CI 5.2–15.1; *p* < 0.01), diabetes remission (OR 3.48; 95% CI 2.02–6.02; *p* < 0.001) with a similar number of long-term complications (OR = 0.19, 95% CI 0.03–1.36; *p* = 0.10). Short-term complications were inferior in the subgroup of patients undergoing SADI-S with < 50 kg/m^2^ of BMI (OR 0.45, 95% CI 0.33 to 0.61; *p* < 0.01) as well as hospital stay (MD = –0.69; 95% CI –1.03 to –0.36, *p* < 0.01) and severe complications (OR = 0.44, 95% CI 0.25–0.80; *p* = 0.01).

**Conclusions:**

This meta-analysis suggests that SADI-S may offer advantages over RYGB in terms of weight loss, diabetes remission, and safety profile.

**Supplementary Information:**

The online version contains supplementary material available at 10.1007/s11695-025-08092-0.

## Introduction

Obesity has become a major global public health challenge, with its prevalence projected to increase substantially in the coming decades[1–3]. Given its strong association with type 2 diabetes (T2D) and other comorbidities, severe obesity imposes a significant clinical and economic burden worldwide [4]. Metabolic bariatric surgery remains the most effective strategy to achieve durable weight loss and remission of obesity-related metabolic diseases [2, 5–7].

Among the commonly performed procedures, Roux-en-Y gastric bypass (RYGB) has traditionally been considered the gold standard due to its balance between efficacy and safety [2, 5, 6]. However, weight regain and T2D relapse continue to pose significant challenges, particularly among patients with more severe forms of obesity [8]. Cooper et al. reported a weight regain of more than 25% from the nadir weight in 36.9% of patients after 6.9 years of follow-up [9], while King et al. observed that over 50% of patients regained more than 15% of their nadir weight within 5 years postoperatively [10]. Moreover, maintaining long-term glycaemic control after RYGB remains a concern. Approximately 50% of patients who achieve diabetes remission (DR) at 1 year experience relapse within 5 years, resulting in a sustained remission rate of only around 30%. These relapses are frequently associated with weight regain and deterioration in lipid profiles beyond the first postoperative year [11–13].

Since its first description in 2007, laparoscopic single-anastomosis duodeno-ileal bypass with sleeve gastrectomy (SADI-S) has gained recognition as a simplified modification of the biliopancreatic diversion with duodenal switch (BPD-DS) [14]. This procedure preserves the robust metabolic and weight-loss effects of BPD-DS while mitigating operative complexity and nutritional risks by using a single anastomosis. Emerging evidence suggests that SADI-S may provide superior weight-loss and metabolic outcomes compared to RYGB, with fewer nutritional complications than BPD-DS—particularly when a common channel of at least 250 cm is maintained [15]. Accordingly, SADI-S is now recognized as an established bariatric procedure by leading organizations, including IFSO and ASMBS [16, 17].

To date, comparative outcomes between SADI-S and RYGB in terms of long-term weight loss, comorbidity remission, and postoperative complications remain inadequately explored [18–20]. While Balamurugan et al. (2023) [21] previously reported comparative outcomes across multiple bariatric techniques, including SADI-S and RYGB, our study provides an updated and focused meta-analytic synthesis with additional clinical endpoints and a larger pooled sample. This systematic review and meta-analysis aim to fill this gap by evaluating and comparing glycaemic control, weight-loss efficacy, and complication rates between these two procedures. The results will offer valuable insights to guide clinical decision-making and optimize surgical strategies for patients with severe obesity.

## Methods

### Search Strategy

**Fig. 1:**
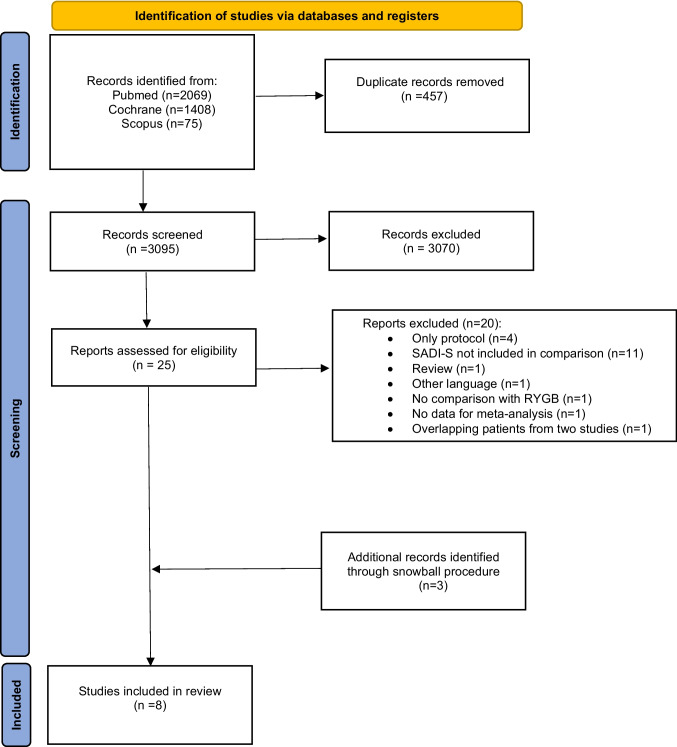
Flow chart of the study selection

A systematic review was conducted in accordance with Cochrane methodology, the Preferred Reporting Items for Systematic Reviews and Meta-Analyses (PRISMA), and the Meta-analysis Of Observational Studies in Epidemiology (MOOSE) guidelines [22–24]. Three electronic databases (PubMed, the Cochrane Library, and Scopus) were systematically searched for studies published in English from inception until May 17, 2025, that compared SADI-S and RYGB in terms of weight loss, remission of obesity-related medical problems, and postoperative complications. The full search strategy is presented in Fig. 1. The following keywords were used: “SADI-S” or “single anastomosis duodeno-ileal bypass with sleeve gastrectomy,” and “RYGB” or “Roux-en-Y gastric bypass” or “gastric bypass.” All possible keyword combinations were explored, and a manual cross-reference check was conducted using the reference lists of included studies and relevant reviews. The study protocol was registered and updated in PROSPERO (CRD42024532785).

### Study Selection and Inclusion Criteria

Eligible designs included randomized controlled trials, prospective controlled studies, matched cohort studies, retrospective cohort studies, and case–control studies. Reviews, editorials, case reports, and prior meta-analyses were excluded.

Studies were included if they met the following criteria: (1) compared SADI-S and RYGB as primary bariatric procedures; (2) reported weight loss in terms of total weight loss (TWL), excess weight loss (EWL), or changes in body mass index (BMI); (3) provided data on associated medical problems remission, including type 2 diabetes, hyperlipidaemia, or obstructive sleep apnoea (OSA), with HbA1c values reported pre- and postoperatively for diabetes; (4) reported on postoperative complications, length of hospital stay, or operative time. For the analysis of weight loss, associated medical problem remission, and long-term complications, a minimum follow-up of 2 years was required to mitigate selection bias. Studies with a mean or median BMI < 35 kg/m^2^ were excluded.

Two authors (LS and PO) independently screened titles and abstracts. Full texts were reviewed in duplicate and blinded, and disagreements were resolved by consensus. Exclusion reasons were documented in detail (Supplementary Material S.13).

### Data Extraction and Definition of Outcomes

Baseline characteristics, preoperative BMI, and the number of patients with type 2 diabetes at baseline were extracted and summarized in Table 1.

**Table 1:** Characteristics of each study and comparison of outcomes between SADI-S and RYGB

Study (year)	Design of study	Follow Up (years)	Nº	Group	Demographic parameters	Weight loss parameters	Associated medical problems remission	Surgery related outcomes
Hage et al.(2024)	USA(Jan 2008-Sept 2023)Retrospective multicenter cohort study	3.6	625	SADI-S(CC: 250-300 cm)	405 women, 220 menMean age 41.9 y ± 11.4Pre-DM2 169 (27%)	Pre-BMI Mean 56.8 ± 6.1Post-BMI Mean 34.3 ± 7.6TWL (%) Mean 40.4 ± 9.1EWL(%) -	DM2r 99 (58.5%)HTAr 129 (43.1%)	Short term complications 10 (1.6%)Long term complications 19 (3%)Severe complications –Hospital stay (days) 2.1 ± 1.9Operative time (mins) 112.2 ± 61.9
			343	RYGB(BPL:50-75 cm/AL:100-150 cm)	256 women, 87 menMean age 44.9 y ± 13.1 Pre-DM2 117 (34%)	Pre-BMI Mean 57.6 ± 7Post-BMI Mean 41.8 ± 8.6TWL (%) Mean 25.8 ± 12.6EWL(%) -	DM2r 44 (37.2%)HTAr 39 (20.3%)	Short term complications 10 (3%)Long term complications 35 (10.2%)Severe complications -Hospital stay (days) 2.7 ± 3.1Operative time (mins) 163.6 ± 58.2
Surve et al.(2020)	USA(Jan 2007 – Dec 2010)Retrospective matched single center cohort study	7	61	SADI-S(CC: 300 cm)	49 women, 12 menMean age 49.1 y ± 14.2Pre-DM2 30 (49.1%)	Pre-BMI Mean 47.8 ± 8.1 Post-BMI Mean 29.1 ± 5.9 TWL (%) Mean 37.8 ± 4.9EWL(%) Mean 73.5 ± 9.7	-	Short term complications 6 (9.8%)Long term complications 12 (19.6%)Severe complications 5 (8.2%)Hospital stay (days) 2 ± 1Operative time (mins) -
			61	RYGB(BPL: 30 cm/AL: 150 cm)	49 women, 12 menMean age 44.3 y ± 13.2Pre-DM2 22 (36%)	Pre-BMI Mean 48.1 ± 8.5Post-BMI Mean 34.1 ± 3.9TWL (%) Mean 32.5 ± 7.5EWL(%) Mean 62.4 ± 12.3	-	Short term complications 6 (10%)Long term complications 38 (62.2%)Severe complications 42 (68.8%)Hospital stay (days) 2.7 ± 0.9Operative time (mins) -
Enochs et al.(2019)	USA(Apr 2014-Oct 2015)Retrospective single center cohort study	2	160	SADI-S(CC: 300 cm)	125 women, 35 menMean age 46 y ± 10Pre-DM2 102 (63.7 %)	Pre-BMI Mean 48.2 ± 8.1 Post-BMI Mean 27.9 ± 4.6TWL (%) –EWL(%) Mean 88.6 ± 20	DM2r 39 (38.2%)	-
			270	RYGB(BPL: 50 cm/AL: 75 cm)	211 women, 59 menMean age 49 y ± 11 Pre-DM2 149 (55.1%)	Pre-BMI Mean 47 ± 7.5Post-BMI Mean 30.3 ± 6.2TWL (%) – EWL(%) Mean 78.3 ± 22.6	DM2r 23 (15.4%)	-
Cottam et al.(2018)	USA(2010–2016)Retrospective single center cohort study	3	341	SADI-S(CC: 300 cm)	218 women, 123 menMean age 47.2 y ± 13.6Pre-DM2 140 (41%)	Pre-BMI Mean 49.6 ± 9Post-BMI Mean 29.3 ± 8TWL (%) - EWL(%) Mean 86.1 ± 27.5	DM2r 93 (66.4%)	Short term complications 67 (19.6%)Long term complications 70 (20.5%)Severe complications 28 (8.2%)Hospital stay (days) - Operative time (mins) -
			457	RYGB(BPL: 30 cm/AL: 150 cm)	365 women, 92 menMean age 44.5 y ± 12.8Pre-DM2 146 (31.9%)	Pre-BMI Mean 48.3 ± 9.2Post-BMI Mean 31.6 ± 7.8TWL (%) Mean – EWL(%) Mean 79.9 ± 25.1	DM2r 69 (47.2%)	Short term complications 163 (36%)Long term complications 267 (58.4%)Severe complications 84 (18.3%)Hospital stay (days) - Operative time (mins) -
Torres et al.(2017)	Spain(2007-2017)Retrospective single center cohort study	3	106	SADI-S(CC: 250-300 cm)	62 women, 44 menMean age 46.2 y ± 11.7Pre-DM2 97 (91%)	Pre-BMI Mean 45.9 ± 6.4 Post-BMI Mean 28.1 ± 4.6TWL (%) Mean 38.7 ± 10.7EWL(%) Mean 87.6 ± 21.9	DM2r 74 (76.2%)	-
			149	RYGB(BPL: 50-75 cm/AL: 150 cm)	96 women, 53 menMean age 48.5 y ± 10.1Pre-DM2 97 (65 %)	Pre-BMI Mean 42.1 ± 6.1Post-BMI Mean 29.9 ± 5.5TWL (%) Mean 28.7 ± 9.7EWL(%) Mean 72.3 ± 25.7	DM2r 54 (55.6%)	-
Arrue del cid et al.(2019)	Spain(Jul 2009 – Jun 2014)Retrospective propensity score matched single center cohort study	5	68	SADI-S(CC: 250-300 cm)	46 women, 22 menMean age 47.1 y ± 11.4Pre-DM2 43 (63.2%)Pre-HTA 37 (54.4%)	Pre-BMI Mean 45.4 ± 5.2Post-BMI - TWL (%) Mean 39.6EWL(%) -	DM2r 34 (79%)HTAr 21 (57%)	Short term complications 7 (10.2%)Long term complications -Severe complications 3 (4.4%)Hospital stay (days) 7.1 ± 8.1Operative time (mins) -
			68	RYGB(BPL: 50-75 cm/AL: 150 cm)	45 women, 23 menMean age 46.7 y ± 10.2 Pre-DM2 23 (33.8%)Pre-HTA 41 (60.2%)	Pre-BMI Mean 44.2 ± 5.3Post-BMI -TWL (%) Mean 28.4EWL(%) -	DM2r 16 (71.4%)HTAr 18 (43%)	Short term complications 13 (19%)Long term complications -Severe complications 3 (4.4%)Hospital stay (days) 7.1 ± 9.6Operative time (mins) -
Sessa et al.(2019)	Italy(Jul 2016-Feb 2018)Retrospective matched single center cohort study	0.83	9	SADI-S(CC: 250 cm)	5 women, 4 menMean age 37.3 y ± 9.5Pre-DM2 0Pre-HTA 6 (66.6%)	Pre-BMI Mean 51.2 ± 8.9 Post-BMI Mean 46.1 ± 7.6TWL (%) -	-	Short term complications 0 Long term complications 0Severe complications 0 Hospital stay (days) 3.7 ± 0.7Operative time (mins) 152.1 ± 30.4
			11	RYGB(BPL: 75 cm/AL: 150 cm)	8 women, 3 menMean age 37.5 y ± 10.8Pre-DM2 0Pre-HTA 7 (63.3%)	Pre-BMI Mean 44.7 ± 3.5Post-BMI Mean 41.7 ± 3.4TWL (%) -	-	Short term complications 0Long term complications 0Severe complications 0Hospital stay (days) 2.6 ± 0.8Operative time (mins) 82.3 ± 31.3
Clapp et al.(2022)	USA(2020)Retrospective propensity score matched multi- center cohort study	30 days	255	SADI-S	183 women, 119 menMean age 42.5 y ± 11.2Pre-DM2 90 (35.2%)Pre-HTA 135 (52.9%)	Pre-BMI Mean 51.7 ± 10Post-BMI - TWL (%) -	-	Short term complications 28 (10.9%)Long term complications -Severe complications 18 (7%)Hospital stay (days) 2.2 ± 2.8 Operative time (mins) 131.5 ± 56.3
			1275	RYGB	890 women, 385 menMean age 42.7 y ± 11.2Pre-DM2 442 (34.6%)Pre-HTA 695 (54.5%)	Pre-BMI Mean 51.7 ± 10Post-BMI -TWL (%) -	-	Short term complications 60 (5%)Long term complications -Severe complications 25 (1.9%)Hospital stay (days) 1.6 ± 1.5Operative time (mins) 116.4 ± 54.8

*SADI-S *Single anastomosis duodeno-ileal bypass with sleeve gastrectomy; *RYGB *roux-en-y gastric bypass; *CC *common channel; *BPL *biliopancreatic limb; *AL *Alimentary limb; *pre *pre-surgery; *post *post-surgery; *DM2 *diabetes mellitus type 2; *HTA *hypertension; *BMI *body mass index; *TWL *total weight loss; *EWL *excess weight loss; *DM2r *Diabetes mellitus type 2 remission; *HTAr *hypetension remission

Outcomes were defined as follows:

- Weight loss outcomes
  - Postoperative BMI (kg/m^2^).
  - Total weight loss (TWL): percentage of weight lost from baseline weight.
  - Excess weight loss (EWL): percentage of weight lost in relation to excess weight above ideal body weight.

- Associated medical problems remission:
-
  - Diabetes remission: HbA1c < 6.5% without antidiabetic medications (number of patients/%).
  - Hypertension remission: blood pressure < 140/90 mmHg without antihypertensive medications (number of patients/%).

- Postoperative complications
  - Number of patients with short term complications (< 30 days). Complications included nausea, vomiting, abdominal pain, intestinal obstruction, incisional hernia, haematoma, haemorrhage, internal hernia, ileus, leak, perforation, constipation, diarrhoea, thrombosis, wound infection, cholecystitis, and ketoacidosis.
  - Number of patients with long term complications (> 30 days). Complications included nausea, vomiting, abdominal pain, ulcer, constipation, diarrhoea, gastroesophageal reflux, dumping syndrome, renal failure, stricture, dysphagia, leak, haemorrhage, intestinal obstruction, internal hernia, ventral hernia, hiatal hernia, fistula, candy cane from the roux limb, malnutrition, perforation, adhesions, pancreatitis, thrombosis, weight loss failure, oesophageal spasms, abscess, and stenosis.
  - Severe complications categorized as Clavien-Dindo ≥ IIIb [25].

- Additional variables included:
  - Length of hospital stay (days).
  - Operative time (minutes).

### Study Objectives

The primary objective was to compare SADI-S and RYGB in terms of weight loss and postoperative complications. The secondary objectives were to assess differences in diabetes and hypertension remission. Operative time and hospital stay were also compared.

### Statistical Analysis

A meta-analysis was performed to estimate pooled effect sizes. For dichotomous outcomes (e.g. complications, diabetes, or hypertension remission), odds ratios (ORs) with 95% confidence intervals (CIs) were calculated. For continuous outcomes (e.g. BMI, TWL, EWL, operative time, hospital stay), mean differences (MDs) with 95% CIs were computed.

For weight loss, associated medical problems remission, and long-term complications, only studies with ≥ 2 years of follow-up were included. In contrast, all eligible studies were used to analyse short-term complications, operative time, and hospital stay, as these are independent of follow-up duration.

All analyses were conducted using a random-effects model (restricted maximum likelihood), accounting for between-study variability. Forest plots were used to visualize individual and pooled estimates. Outcomes on different scales were standardized as necessary.

Heterogeneity was assessed using the *I*^2^ statistic, with thresholds of < 25% (none), 25–50% (low), 50–75% (moderate), and > 75% (high). Subgroup, sensitivity, and meta-regression analyses were conducted when applicable to explore and adjust for heterogeneity.

All analyses were performed using Stata version 19 [StataCorp, College Station, TX, USA], ensuring reproducibility and transparency.

### Risk of Bias Assessment

The risk of bias was assessed independently by LS and PM using the Newcastle–Ottawa Scale (NOS) [26], evaluating selection, comparability, and outcome across eight domains. Studies were categorized as low (< 4), moderate (4–6), or high (≥ 7) quality. Discrepancies were resolved by consensus.

Studies were assessed according to the outcomes relevant to their follow-up duration: those with < 2 years of follow-up were assessed for short-term outcomes; those with ≥ 2 years were evaluated for long-term outcomes. This ensured consistency in quality assessment relative to the timeframe of the reported data (Supplementary Material S.12).

Publication bias was evaluated using funnel plots, the Egger’s test (*p* < 0.10), and the “metafunnel” and “metabias” functions in Stata. Results are detailed in Supplementary Materials S.1–S.11.

## Results

### Study Selection

The initial search strategy yielded 3552 relevant articles. Following the removal of duplicates and abstract screening, 25 articles were retained for full-text review. Following this, studies that did not meet the predefined criteria were excluded from the final analysis (Fig. 1; Supplementary material S.13). In the end, eight articles [27–34] met the inclusion criteria (Table 1). The quality assessment of the studies included is shown in the Supplementary material S.12.

### Characteristics of Study Population

Among the 4259 patients included in the analysis, 1625 (38.1%) underwent SADI-S. The studies were divided into two groups based on follow-up duration: six studies [27–31, 33] with more than 2 years of follow-up and two studies [32, 34] with less than 2 years. For long-term outcomes, such as weight loss, remission of associated medical problems, and long-term complications, only the six studies [27–31, 33] with extended follow-up were included. These studies encompassed a total of 2709 patients, with 1361 (50.2%) in the SADI-S group and 1348 (49.7%) in the RYGB group, and an average follow-up period of 3.93 years (1.79).

In contrast, all eight studies [27–34], regardless of follow-up duration, were included in the analysis of short-term outcomes, such as operative time, hospital stay, and short-term complications, since these outcomes are not influenced by follow-up length.

### Weight Loss Outcomes

#### Body Mass Index

The preoperative BMI was statistically significantly higher in the SADI-S group (studies: 8 [27–34]; MD 1.35; 95% CI 0.40–2.29; *p* = 0.01; *I*^2^ = 63.6; Supplementary material S.1), and the postoperative BMI at longest follow-up (3.72 years (0.85)) was significantly lower (studies: 5 [27–31]; MD − 3.79; 95% CI − 5.94, − 1.65; *p* < 0.01; *I*^2^ = 93.5; Supplementary material S.2).

#### Total Weight Loss (%)

**Fig. 2:**
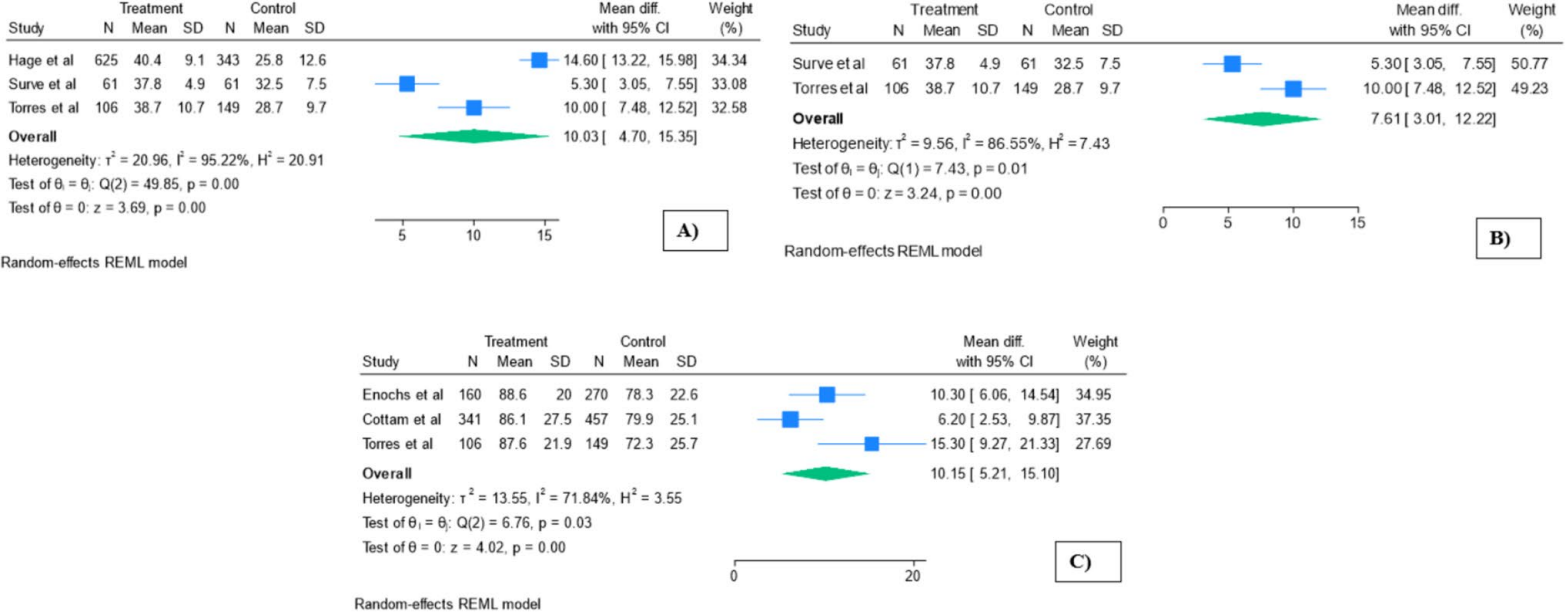
Weight loss outcomes meta-analysis. **A** Total weight loss (%). **B** Total weight loss (%) after sensitivity analysis. **C** Excess weight loss (%). Treatment, SADI-S; control, RYGB

Patients undergoing SADI-S had a statistically significant higher total weight loss rate at a mean follow-up of 4.65 years (0.88) (studies: 3 [27, 28, 31]; MD 10.03; 95% CI 4.7–15.35; *p* < 0.001; *I*^2^ = 95.2%; Fig. 2A; Supplementary material S.3). A sensitivity analysis excluding Hage et al. [27], identified as a potential outlier due to markedly higher baseline BMI, was conducted. After removal, the random-effects meta-analysis of the remaining two studies [28, 31] showed a significant pooled mean difference of 7.61 (95% CI 3.01 to 12.22; *p* < 0.01) (Fig. 2B). However, considerable heterogeneity persisted (*I*^2^ = 86.5%). A random-effects meta-regression including the three studies found no significant association between preoperative BMI and total weight loss (*β* = 0.58; 95% CI –3.60 to 4.75; *p* = 0.787), with no explained variance (*R*^2^ = 0%) and high residual heterogeneity (*I*^2^ = 97.96%; *τ*^2^ = 39.39).

### Excess Weight Loss (%)

Focusing on the percentage of excess weight loss, SADI-S offers significantly better results than the RYGB (studies 3 [29–31]; MD 10.15; 95% CI 5.2–15.1; *p* < 0.01; *I*^2^ = 71.84%; Fig. 2C, Supplementary material S.4). A random-effects meta-regression using preoperative BMI difference as a covariate explained 59.4% of the between-study heterogeneity (*R*^2^ = 59.4%), and the residual heterogeneity was not statistically significant (*Qₑ* = 2.35, *p* = 0.125).

### Associated Medical Problem Remission

#### Diabetes Mellitus Type 2

**Fig. 3:**
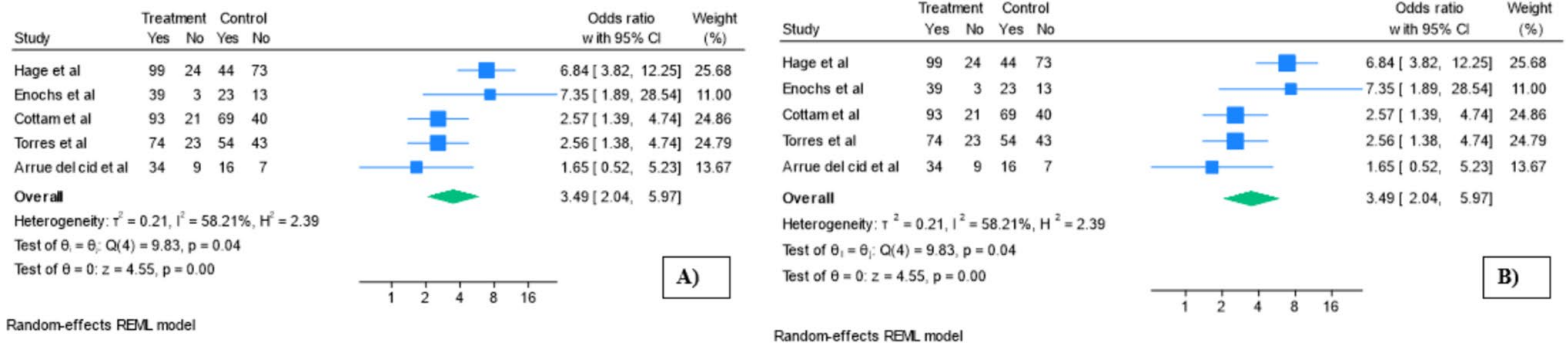
Diabetes remission meta-analysis. **A** Diabetes remission. **B** Diabetes remission after sensitivity analysis. Treatment, SADI-S; control, RYGB

Diabetes remission was significantly improved in the SADI-S group (studies: 5 [27, 29–31, 33]; OR 3.48; 95% CI 2.02–6.018; *p* < 0.01; *I*^2^ = 59.3%; Fig. 3A, Supplementary material S.5) at a mean follow-up of 3.93 years (0.73). A sensitivity meta-analysis was conducted excluding the study by Hage et al. [27], which was identified as having a notably higher baseline prevalence of type 2 diabetes in the RYGB group compared to the SADI-S group, in contrast to the other studies [29–31, 33] (Fig. 3B). The random-effects model yielded a pooled odds ratio of 2.66 (studies 4[25–27, 29] 95% CI 1.80 to 3.93, *p* < 0.01, *I*^2^ = 0%) in favour of SADI-S.

#### Hypertension

No differences were found in hypertension remission (studies: 2 [27, 33]; OR 3.8; 95% CI 0.82–17.64; *p* = 0.08; *I*^2^ = 92.8%; Supplementary material S.6).

### Postoperative Complications

#### Short-Term Complications

Both groups had similar risk of short-term complications (studies: 5 [27, 30, 32–34]; OR 0.77; 95% CI 0.34–1.77; *p* = 0.54; *I*^2^ = 84.72%; Supplementary material S.7).

**Fig. 4:**
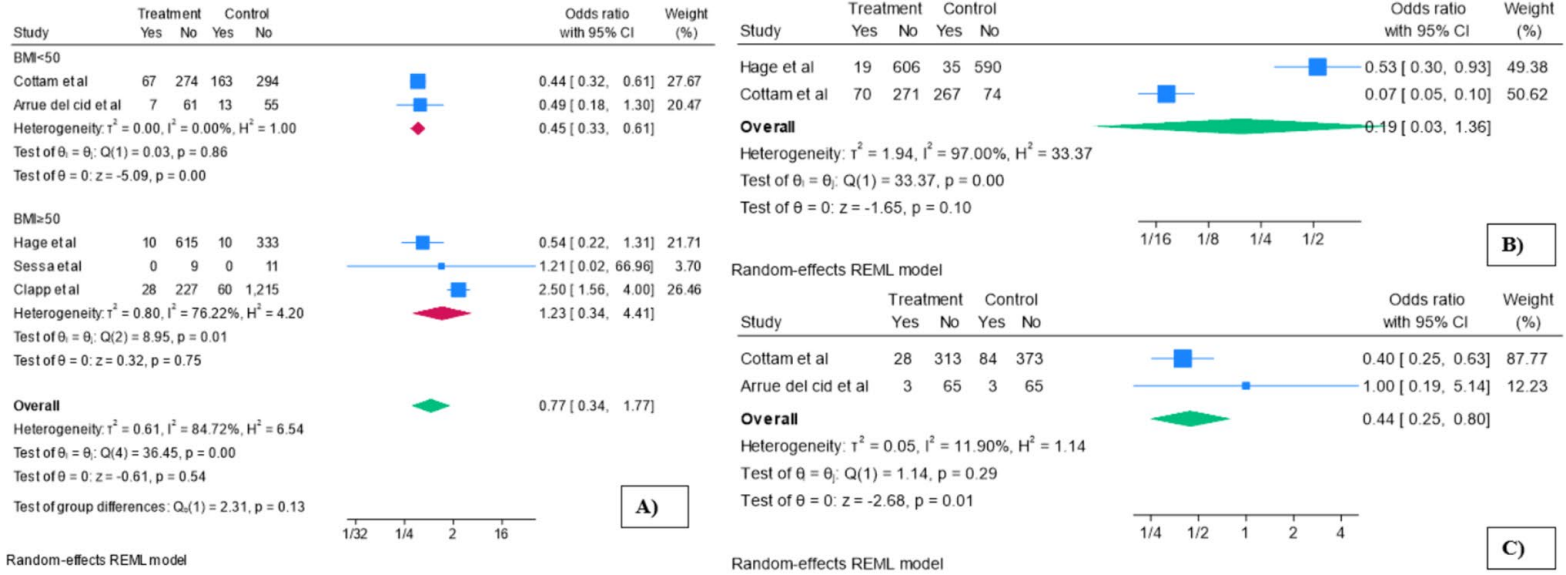
Postoperative complications. **A** Subgroup analysis of short-term complications. **B** Long-term complications. **C** Sensitivity analysis of severe complications (BMI < 50 kg/m^2^). Treatment, SADI-S; control, RYGB

A subgroup meta-analysis was performed to assess the influence of preoperative BMI (< 50 vs. ≥ 50 kg/m^2^). The analysis revealed a significant difference in short-term complication rates between the groups with BMI < 50 kg/m^2^ and those with BMI ≥ 50 kg/m^2^. In the subgroup of patients with BMI < 50 kg/m^2^ [28, 30, 33], the SADI-S group demonstrated a lower incidence of short-term complications compared to the RYGB group, with an OR of 0.45 (95% CI 0.33 to 0.61, *p* < 0.01; *I*^2^ = 0) (Fig. 4A). In contrast, for patients with BMI ≥ 50 kg/m^2^ [27, 32, 34], the SADI-S group did not show a significant difference in complication rates compared to RYGB, with an OR of 1.233 (95% CI 0.345 to 4.413, *p* = 0.75). Furthermore, a meta-regression adjusting for the difference in preoperative BMI failed to show a significant association with short-term complication rates (*β* = –0.30; *p* = 0.31), and it did not explain the between-study heterogeneity (*R*^2^ = 0%).

#### Long-Term Complications

The pooled analysis of long-term complications showed a non-significant reduction in the SADI-S group compared to RYGB (studies: 3 [27, 28, 30]; OR = 0.19, 95% CI 0.03–1.36; *p* = 0.10), with substantial heterogeneity across studies (*I*^2^ = 97%) (Fig. 4B).

#### Severe Complications

The meta-analysis of severe complications showed no statistically significant difference between groups (OR = 1.15, 95% CI 0.32–4.18; *p* = 0.83), with substantial heterogeneity across studies (*I*^2^ = 85.8%). After excluding the two outlier studies [32, 34] reporting a mean preoperative BMI greater than 50 kg/m^2^, a random-effects sensitivity meta-analysis was conducted on the remaining two studies [28, 30, 33] assessing severe complications. The incidence of severe complications was significantly lower in the SADI-S group (studies 2 [30, 33] OR = 0.44, 95% CI 0.25–0.80; *p* = 0.01), with low heterogeneity (*I*^2^ = 11.9%) (Fig. 4C).

### Operative Duration

The operative time was similar in both groups (studies: 3 [27, 32, 34]; MD 10.2; 95% CI − 57.9–78.4; *p* = 0.7; *I*^2^ = 99.2%; Supplementary material S.10). A meta-regression adjusting for preoperative BMI was made, not explaining any of the heterogeneity observed.

### Hospital Stay

Patients had an equivalent length of stay when comparing both interventions (studies: 5 [27, 28, 32–34]; MD 0.07; 95% CI − 0.69–0.822; *p* = 0.87; *I*^2^ = 94.1%; Supplementary material S.11). A subgroup meta-analysis comparing mean hospital stay between SADI-S and RYGB according to preoperative BMI revealed contrasting results. In studies with BMI < 50 kg/m^2^ [28, 33], SADI-S was associated with a significantly shorter hospital stay (MD = –0.69; 95% CI –1.03 to –0.36, *p* < 0.01), whereas in studies with BMI ≥ 50 kg/m^2^ [27, 32, 34], the difference was not statistically significant (MD = 0.33; 95% CI –0.65 to 1.31).

### Quality Assessment and Risk of Bias Assessment

The studies were categorized into two groups based on follow-up duration: those reporting short-term outcomes and those focused on long-term outcomes. Each study was carefully evaluated for the outcomes relevant to its follow-up category, and methodological quality was assessed using the Newcastle–Ottawa Scale (NOS), with all studies scoring 8 for the selected outcomes (Supplementary material S.12). Although the studies by Arrue del Cid et al.[33] and Torres et al. [31] originated from the same institution, both were retained since their reported outcomes were distinct, except for diabetes remission. Sensitivity analyses were conducted to assess potential duplication bias, confirming that the inclusion of both studies did not affect the overall findings.

Regarding the studies by Cottam et al. [30] and Surve et al. [28], author correspondence revealed a possible minimal overlap in patient recruitment during a brief time window. To address this, for outcomes reported in both studies, only the data from Cottam et al. [30] were used, as it corresponds to the original cohort and includes a larger patient sample.

### Publication Bias

The Egger’s test did not reveal statistical evidence of publication bias, with the exception of EWL (*p* < 0.1). However, given the limited number of studies included, the possibility of undetected bias cannot be entirely ruled out (Supplementary material S.1-S.11).

## Discussion

This systematic review and meta-analysis provides an updated and comprehensive comparison of SADI-S and RYGB in terms of weight loss, diabetes remission, and postoperative safety. Based on data from over 4600 patients [27–34], SADI-S was associated with significantly greater TWL, higher EWL, and improved DR compared to RYGB.

These findings are notable considering that patients undergoing SADI-S had higher baseline BMIs and more severe metabolic profiles. The superior metabolic outcomes may be explained by the unique anatomical features of SADI-S, pylorus preservation [14], longer biliopancreatic limb [14, 35], and resulting changes in gut microbiota [36]—all of which contribute to enhanced glycaemic control and weight loss.

Regarding postoperative safety, SADI-S demonstrated significantly lower rates of short-term and severe complications, particularly in patients with a BMI < 50 kg/m^2^. These advantages may be partly attributed to the preservation of the pylorus, which reduces risks of marginal ulcers and internal hernias [14], and to the avoidance of mesenteric defects. Additionally, hospital stay was shorter in this subgroup.

Despite these benefits, SADI-S has not yet achieved widespread adoption. Its more demanding technical requirements, particularly the management of the duodenal stump and proximity to vital structures, likely contribute to its slower integration into mainstream practice [34, 35].

Nutritional deficiencies, a common concern in malabsorptive procedures, were difficult to evaluate due to limited and heterogeneous data [27–34]. However, available evidence suggests that when performed with a common channel of 250–300 cm and appropriate supplementation, SADI-S can offer an acceptable long-term nutritional profile [19–21, 35]. Compared to other hypoabsorptive procedures, such as duodenal switch (DS) and one-anastomosis gastric bypass (OAGB), SADI-S offers a more favourable balance of efficacy and safety. DS is associated with high nutritional risk due to its complexity [37], while OAGB raises concerns about bile reflux and long-term deficiencies [38]. SADI-S may overcome these limitations by combining effective weight loss with a simpler and safer anatomical configuration [35].

This study has several limitations. All included studies were observational in nature, and the variability in follow-up duration likely contributed to the substantial heterogeneity observed. Although we addressed this through sensitivity and subgroup analyses, these adjustments reduced the overall sample size and, consequently, the statistical power of the meta-analyses. Furthermore, considerable procedural heterogeneity was present across studies, particularly with respect to the lengths of the biliopancreatic limb. While most SADI-S studies reported relatively homogeneous configurations (typically 250–300 cm common channel), greater variation was observed among biliopancreatic limb in RYGB cohorts, which may have influenced outcomes related to weight loss, nutritional status, and complication rates. These findings should therefore be interpreted with caution. Further high-quality RCTs are warranted to confirm these associations and reinforce the current evidence base. Notably, a multicentre RCT is currently underway and may provide definitive data on the long-term safety and efficacy of SADI-S compared to RYGB [39].

In conclusion, our findings support SADI-S as a safe and effective bariatric option, especially for patients with higher BMI or severe metabolic disease. Compared to RYGB, SADI-S appears to offer superior weight loss and metabolic outcomes, with a favourable safety profile. Future randomized trials with long-term follow-up are needed to validate these results and to refine patient selection criteria.

## Conclusion

This meta-analysis suggests that SADI-S may offer advantages over RYGB in terms of weight loss, diabetes remission, and safety profile.

## Supplementary Information

Below is the link to the electronic supplementary material.

ESM 1 (DOCX 314 KB)

## Data Availability

No datasets were generated or analysed during the current study.
