# Supplementary material for: Comparison Between Single Anastomosis Duodeno-Ileal Bypass with Sleeve Gastrectomy (SADI-S) and Roux-En-Y Gastric Bypass (RYGB) in Terms of Weight Loss, Associated Medical Problems Remission, and Complications: A Systematic Review with Meta-Analysis
Source: Obes Surg. 2025 Jul 22;35(9):3577–90. doi: 10.1007/s11695-025-08092-0 (PMC12457536; doi:10.1007/s11695-025-08092-0)
Supplement: Supplementary file 1 — (DOCX 314 KB) [file 11695_2025_8092_MOESM1_ESM.docx]

**TABLE OF CONTENTS**

**S.1 Pre-BMI 3**

**S.2 Post-BMI 4**

**S.3 Total weight loss (TWL) 5**

**S.4 Excess weight loss 7**

**S.5 Diabetes Mellitus remission 8**

**S.6 Hypertension remission 10**

**S.7 Short-term postoperative complications 11**

**S.8 Long-term postoperative complications 13**

**S.9 Severe complications (Clavien-Dindo >3) 14**

**S.10 Operative duration 16**

**S.11 Hospital stay 17**

**S.12. Assessment of methodological quality 19**

**S.13. Studies excluded from the systematic review 20**

**Appendix 1. Comparative Group Designation:**

**SADI-S (Treatment) / RYGB (Control)**

# S.1 Pre-BMI

- 1. Forest plot showing results of pre-BMI (kg/m²) mean difference metanalysis

- 1. Funnel plot and Egger’s test showing publication bias; Mean difference pre-BMI (kg/m²) (X-axis) with it is standard error (Y-axis)

# S.2 Post-BMI

- 1. Forest plot showing results of post-BMI (kg/m²) mean difference meta-analysis

- 1. Funnel plot and Egger’s test showing publication bias; Mean difference post-BMI (kg/m²) (X-axis) with it is standard error (Y-axis)

# S.3 Total weight loss (TWL)

3.1. Forest plot showing results of TWL (%) mean difference metanalysis

3.2. Forest plot showing results of TWL (%) sensitivity analysis excluding Hage et al. (outlier due to baseline BMI>50)

1. Funnel plot and Egger test showing publication bias; Mean difference TWL (X-axis) with it is standard error (Y-axis)

# S.4 Excess weight loss

4.1. Forest-plot showing results of excess weight loss meta-analysis

4.2. Funnel plot and Egger’s test showing publication bias; Mean difference EWL (X-axis) with it is standard error (Y-axis)

# S.5 Diabetes Mellitus remission

5.1. Forest plot showing results of diabetes mellitus remission meta-analysis

5.2. Forest plot showing results of diabetes mellitus remission sensitivity analysis excluding Hage et al. (Outlier study identified due to a higher baseline prevalence of diabetes in the RYGB group, opposite from the other studies)

- 1. Funnel plot and Egger’s test showing publication bias; diabetes mellitus remission (log OR)(X-axis) with it is standard error (Y-axis)

# S.6 Hypertension remission

6.1. Forest plot showing results of Hypertension remission metanalysis

6.2. Funnel plot showing publication bias; Hypertension remission (log OR)(X-axis) with it is standard error (Y-axis)

# S.7 Short-term postoperative complications

- 1. Forest plot showing results of short-term postoperative complications metanalysis

- 1. Forest plot showing results of subgroup analysis of short-term postoperative complications separating by BMI (</> 50 Kg/m^2^)

- 1. Funnel plot and Egger’s test showing publication bias; short-term postoperative complications (log OR)(X-axis) with it is standard error (Y-axis)

# S.8 Long-term postoperative complications

- 1. Forest plot showing results of long-term postoperative complications metanalysis

- 1. Funnel plot showing publication bias; long-term postoperative complications (log OR)(X-axis) with it is standard error (Y-axis)

# S.9 Severe complications (Clavien-Dindo >3)

- 1. Forest plot showing results of severe complications metanalysis

- 1. Forest plot showing results of sensitivity analysis excluding outliers with preoperative BMI > 50 Kg/m^2^ (Sessa et al. and Clapp et al.)

- 1. Funnel plot and Egger’s test showing publication bias; severe postoperative complications (log OR)(X-axis) with it is standard error (Y-axis)

# S.10 Operative duration

10.1. Forest plot showing results of mean difference operative duration (minutes) metanalysis

- 1. Funnel plot showing publication bias; mean difference operative duration (minutes) with it is standard error (Y-axis)

# S.11 Hospital stay

11.1. Forest plot showing results of hospital stay (days) metanalysis

11.2. Forest plot showing results of subgroup analysis of hospital stay separating by BMI (</> 50 Kg/m^2^)

- 1. Funnel plot showing publication bias; mean difference in hospital stay (days) with it is standard error (Y-axis)

# S.12. Assessment of methodological quality

| **Table 3.** Newcastle-Ottawa score for the included studies | | | | | | | | | |
| --- | --- | --- | --- | --- | --- | --- | --- | --- | --- |
| **First author, year** | Representativeness of cohort | Selection of non-exposed cohort | Ascertainment of exposure | Demonstration that outcome of interest was not present at start of study | Comparability of cohorts on the basis of the design or analysis | Assessment of outcome | Was follow-up long enough for outcomes to occur | Adequacy of follow up of cohorts | Total score |
| **Hage et al (2024)** | ★ | ★ | ★ | ★ | ★ | ★ | ★ | ★ | **8** |
| **Surve et al (2020)** | ★ | ★ | ★ | ★ | ★ | ★ | ★ | ★ | **8** |
| **Enochs et al (2019)** | ★ | ★ | ★ | ★ | ★ | ★ | ★ | ★ | **8** |
| **Cottam et al (2018)** | ★ | ★ | ★ | ★ | **★** | ★ | ★ | ★ | **8** |
| **Torres et al (2017)** | ★ | ★ | ★ | ★ | ★ | ★ | ★ | **★** | **8** |
| **Arrue del CId et al (2019)** | ★ | ★ | ★ | ★ | **★** | ★ | ★ | ★ | **8** |
| **Verhoef et al (2022)*** | ★ | ★ | ★ | ★ | ★ | ★ | ★ | ★ | **8** |
| **Clapp et al (2022)*** | ★ | ★ | ★ | ★ | ★ | ★ | ★ | ★ | **8** |
| **Sessa et al (2019)*** | ★ | ★ | ★ | ★ | ★ | ★ | ★ | ★ | **8** |

*****This studies were evaluated just for preoperative variables, short-term complications, operative duration and hospital stay.

**S.13. Studies excluded from the systematic review**

| **Table 4.** Studies excluded from the systematic review | | |
| --- | --- | --- |
| **Study** | **PMID/DOI** | **Cause of exclusion** |
| Prospective multicentre randomised trial comparing the efficacy and safety of single-anastomosis duodeno-ileal bypass with sleeve gastrectomy (SADI-S) versus Roux-en-Y gastric bypass (RYGB): SADISLEEVE study protocol | **35414539** | Only protocol |
| Comparison of Efficacy and Safety Between Roux-en-Y Gastric Bypass (RYGB) vs One Anastomosis Gastric Bypass (OAGB) vs Single Anastomosis Duodeno-ileal Bypass with Sleeve Gastrectomy (SADI-S): a Systematic Review of Bariatric and Metabolic Surgery | **34981238** | Review |
| [Comparative results of various methods of surgical treatment of severe forms of metabolic syndrome]. | **22950270** | SADI-S not included |
| Evaluation of the Efficacy of Single Anastomosis Sleeve Ileal (SASI) Bypass for Patients with Morbid Obesity: a Multicenter Study | **31734889** | No comparison with RYGB |
| Comparison of short- and long-term outcomes of bariatric surgery methods: A retrospective study. | **36197162** | SADI-S not included |
| Bariatric surgery: effects on the metabolic complications of obesity. | **22287091** | SADI-S not included |
| Single anastomosis duodenal switch (SADI-S) versus Roux-en-y gastric bypass-defining a new gold standard in metabolic surgery | - | Only protocol |
| Outcomes of SADI and OAGB Compared to RYGB from the Metabolic and Bariatric Surgery Quality Improvement Program: The North American Experience | **10.1007/s11695-023-07019-x** | No data for metaanalisis |
| A Comparison of the Bariatric Procedures that Are Performed in the Treatment of Super Morbid Obesity. | **28451928** | SADI-S not included |
| Trends in Utilization and Relative Complication Rates of Bariatric Procedures. | **31012048** | SADI-S not included |
| Bariatric surgery and prevention of cardiovascular events and mortality in morbid obesity: mechanisms of action and choice of surgery. | **25770762** | SADI-S not included |
| Morbid obesity treatment by SADI-S: a multi-center randomized controlled clinical trial | **32873678** | Only protocol |
| Long-Term Outcomes of Bariatric and Metabolic Surgery in Japan: Results of a Multi-Institutional Survey. | **27631329** | SADI-S not included |
| **[**Analysis of the 1-year curative efficacy of sleeve gastrectomy, Roux-en-Y gastric bypass, single anastomosis duodenal-ileal bypass with sleeve gastrectomy and biliopancreatic diversion with duodenal switch in patients with super obesity]. | **37709694** | Chinese language |
| Efficacy of Different Procedures of Metabolic Surgery for Type 2 Diabetes in Asia: a Multinational and Multicenter Exploratory Study. | **33523416** | SADI-S not included |
| Single-anastomosis Duodeno Ileal Bypass (SADI) Versus Roux-en-Y Gastric Bypass  NCT03610256 | **-** | Only protocol |
| Comparative Effectiveness and Safety of Bariatric Procedures for Weight Loss: A PCORnet Cohort Study. | **30383139** | SADI-S not included |
| Atherogenic Dyslipidemia Remission 1 Year After Bariatric Surgery | **27988827** | SADI-S not included |
| Comparative Effectiveness of Different Bariatric Procedures in Super Morbid Obesity. | **30251091** | SADI-S not included |
| Patient Selection and 30‑Day Outcomes of SADI‑S Compared to RYGB:  a Retrospective Cohort Study of 47,375 Patients | **https://doi.org/10.1007/s11695-022-06068-y** | Overlapping of patients with other study |
